# Development and Validation of a Questionnaire to Detect Behavior Change in Multiple Advance Care Planning Behaviors

**DOI:** 10.1371/journal.pone.0072465

**Published:** 2013-09-05

**Authors:** Rebecca L. Sudore, Anita L. Stewart, Sara J. Knight, Ryan D. McMahan, Mariko Feuz, Yinghui Miao, Deborah E. Barnes

**Affiliations:** 1 San Francisco Veterans Affairs Medical Center, San Francisco, California, United States of America; 2 Department of Medicine, Division of Geriatrics, University of California San Francisco, San Francisco, California, United States of America; 3 Institute for Health and Aging, University of California San Francisco, San Francisco, California, United States of America; 4 Health Services Research and Development Service, Veterans Administration, Washington, District of Columbia, United States of America; 5 Department of Psychiatry, University of California San Francisco, San Francisco, California, United States of America; 6 Department of Epidemiology and Biostatistics, University of California San Francisco, San Francisco, California, United States of America; Massachusetts General Hospital, United States of America

## Abstract

**Introduction:**

Advance directives have traditionally been considered the gold standard for advance care planning. However, recent evidence suggests that advance care planning involves a series of multiple discrete behaviors for which people are in varying stages of behavior change. The goal of our study was to develop and validate a survey to measure the full advance care planning process.

**Methods:**

The Advance Care Planning Engagement Survey assesses “Process Measures” of factors known from Behavior Change Theory to affect behavior (knowledge, contemplation, self-efficacy, and readiness, using 5-point Likert scales) and “Action Measures” (yes/no) of multiple behaviors related to surrogate decision makers, values and quality of life, flexibility for surrogate decision making, and informed decision making. We administered surveys at baseline and 1 week later to 50 diverse, older adults from San Francisco hospitals. Internal consistency reliability of Process Measures was assessed using Cronbach's alpha (only continuous variables) and test-retest reliability of Process and Action Measures was examined using intraclass correlations. For discriminant validity, we compared Process and Action Measure scores between this cohort and 20 healthy college students (mean age 23.2 years, SD 2.7).

**Results:**

Mean age was 69.3 (SD 10.5) and 42% were non-White. The survey took a mean of 21.4 minutes (±6.2) to administer. The survey had good internal consistency (Process Measures Cronbach's alpha, 0.94) and test-retest reliability (Process Measures intraclass correlation, 0.70; Action Measures, 0.87). Both Process and Action Measure scores were higher in the older than younger group, p<.001.

**Conclusion:**

A new Advance Care Planning Engagement Survey that measures behavior change (knowledge, contemplation, self-efficacy, and readiness) and multiple advance care planning actions demonstrates good reliability and validity. Further research is needed to assess whether survey scores improve in response to advance care planning interventions and whether scores are associated with receipt of care consistent with one's wishes.

## Introduction

Advance care planning is the process of planning for future medical care with the goal of matching patients' preferences with the medical care provided. Traditionally, advance care planning has focused on deciding whether to have medical procedures such as cardiopulmonary resuscitation (CPR) or mechanical ventilation at the end-of-life [1]. Advance care planning has also focused on documenting preferences for medical procedures in a legal document, such as a living will or an advance directive, in case of future decisional incapacity. To date, completion of a written advance directive has been the gold standard for successful advance care planning and the main outcome used to determine whether advance care planning interventions are successful [2]. However, there is growing awareness that advance directives are limited because they do not adequately capture the full range and scope of the multiple behaviors that make up the advance care planning process.

In recent years, advance care planning has been redefined as an ongoing process consisting of several discrete behaviors instead of a one time advance directive document [3], [4]. These advance care planning behaviors include identifying ones' evolving values and goals over the course of health and illness (not just at the end-of-life) and communicating these evolving values and goals to loved ones and physicians over time. Recent research has also demonstrated that behavior change theory plays a role in motivating individuals to engage in advance care planning. Social Cognitive Theory [5], [6] and Behavior Change Theory [6], [7] (the most well established theories for how people change behavior) posit that behavior change requires change in several factors or processes including: (a) knowledge or understanding of the importance of the behavior, (b) contemplation of engaging in the behavior, (c) self-efficacy to complete the behavior, and (d) readiness to complete the behavior. These theories also posit that, based on these factors, individuals then proceed through a series of stages including pre-contemplation, contemplation, and preparation prior to action (changed behavior). With respect to advance care planning behaviors, research has demonstrated that individuals are in varying stages of behavior change for each discrete advance care planning behavior [4].

Therefore, what is needed is a self-report measure that assesses the full range of processes involved in advance care planning (i.e., changes in knowledge, contemplation, self-efficacy and readiness) as well as completion (action) of each discrete advance care planning behavior. This is important because advance care planning interventions may be deemed “unsuccessful” based on the narrow outcome of advance directive completion, yet may actually help people move along the behavior change pathway towards action. In addition, by determining deficits in behavior change factors, interventions could be tailored to facilitate completion of each advance care planning behavior. Given the paucity of surveys that measure the full scope of advance care planning, the goal of this study was to develop and validate a survey designed to quantify the process of behavior change, including knowledge, contemplation, self-efficacy, and readiness, as well as measure completion (action) of multiple advance care planning behaviors.

## Materials and Methods

### Ethics Statement

This study was approved by the institutional Review Boards of the University of California, San Francisco and the San Francisco VA Medical Center. Written informed consent was obtained for all participants. Data will be made freely available upon request.

### Development of the Advance Care Planning Engagement Survey

**Figure 1:**
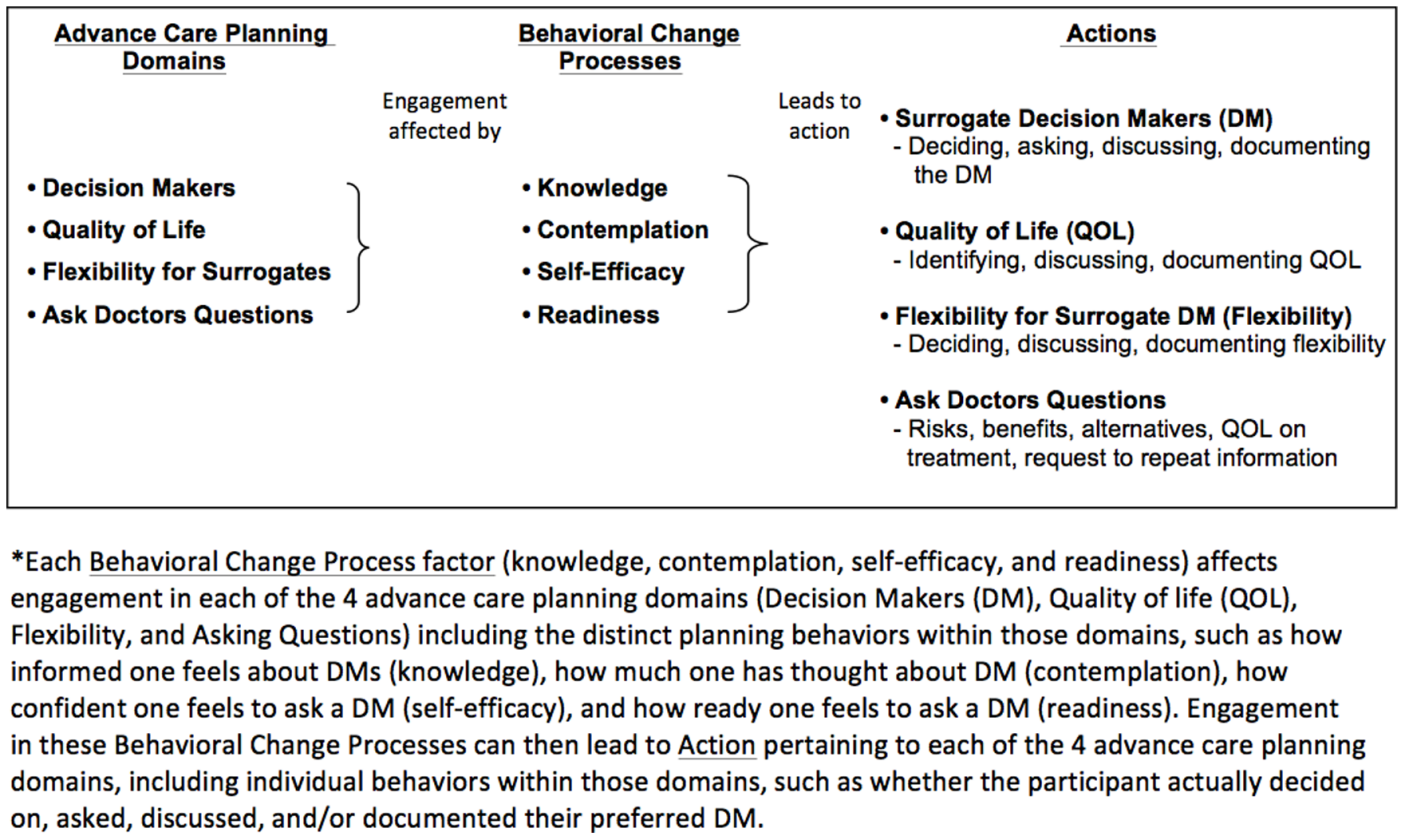
*Each Behavioral Change Process factor (knowledge, contemplation, self-efficacy, and readiness) affects engagement in each of the 4 advance care planning domains (Decision Makers (DM), Quality of life (QOL), Flexibility, and Asking Questions) including the distinct planning behaviors within those domains, such as how informed one feels about DM (knowledge), how much one has thought about DM (contemplation), how confident one feels to ask a DM (self-efficacy), and how ready one feels to ask a DM (readiness). Engagement in these Behavioral Change Processes can then lead to Action pertaining to each of the 4 advance care planning domains, including individual behaviors within those domains, such as whether the participant actually decided on, asked, discussed, and/or documented their preferred DM.

We used several strategies to design the Advance Care Planning Engagement survey. First, we created a conceptual framework of the advance care planning behaviors needed to complete the advance care planning process and to adequately prepare patients for future medical decisions. The conceptual framework was developed through a review of prior research, our own published conceptual model, [1] published data from extensive focus groups with patients and surrogate decisions makers from diverse backgrounds, [8] and input from content experts in advance care planning, behavior change, and survey design and validation. Our conceptual framework of advance care planning includes the following domains: 1) Decision Makers (DM) refers to identifying an appropriate surrogate decision maker, asking that person to assume the responsibility, telling the doctor about the surrogate and documenting the name of the surrogate; 2) Quality of Life (QOL) refers to identifying goals about whether certain health states would make a person's life not worth living and whether they value quality over quantity of life, discussing values and goals with surrogates and clinicians, and documenting their wishes; 3) Flexibility refers to deciding whether and how much flexibility or leeway to grant the surrogate decision maker in making decisions, discussing flexibility with surrogates and doctors, and documenting flexibility; and 4) Asking Questions refers to preparing to ask doctors questions to make informed medical decisions based on identified values and goals. Although we acknowledge that patients are cared for by clinicians from a variety of disciplines, in pilot testing, individuals preferred the term “doctors.” Each advance care planning domain (DM, QOL, Flexibility, Ask Questions) includes several discrete advance care planning behaviors. Based on Social Cognitive Theory [5], [6] and Behavior Change Theory [6], [7] we conceptualized that the behavior change factors of knowledge, contemplation, self-efficacy, and readiness are required to complete each advance care planning behavior (what we define as behavior change processes, Figure 1). Last, we conceptualized the culmination of behavior change as “action” or having completed an individual advance care planning behavior with each advance care planning domain (DM, QOL, Flexibility, Ask Questions, Figure 1).

The Advance Care Planning Engagement Survey contains two sections, “Process Measures” and “Action Measures”. The Process Measures quantify four factors identified from Behavior Change Theory that are needed for people to engage in behavior: knowledge, contemplation, self-efficacy, and readiness, all on a 5-point Likert scale (Table 1). For the Process Measures subscales of knowledge, self-efficacy, and readiness factors, the Likert responses were “not at all, a little, somewhat, fairly, extremely” and for the contemplation subscale the Likert responses were “never, once or twice, a few times, several times, a lot”. As described above and in Figure 1, each behavior change factor (knowledge, contemplation, self-efficacy, and readiness) affects each of the 4 advance care planning domains (DM, QOL, Flexibility, Ask Questions) and individual advance care planning behaviors. Therefore, the Process Measures questions were grouped into subscales for each behavior change factor (knowledge, contemplation, self-efficacy, and readiness) with questions under each factor sub-scale pertaining to behaviors within the advance care planning domains (DM, QOL, Flexibility, Ask Questions). For example, in the subscale of self-efficacy, we asked “How confident are you that today you could…” 1) ask someone to be a medical decision maker (DM); 2) decide whether or not certain health states would make life not worth living (QOL); 3) decide how much flexibility to give your decision maker (Flexibility); and 4) ask doctors questions (Ask Questions). Of note, the knowledge subscale does not ask about QOL as this is a personal determination, not a factual one.

**Table 1:** Advance Care Planning Engagement Survey – Process Measures: Behavior Change Sub-scales, Advance Care Planning Domains, and Questions.

Sub-Scales	Domain*	Questions
**Knowledge** †		**How well informed are you about:**
	DM	- who can be a medical decision maker?
		- what makes someone a good medical decision maker?
		- the types of decisions that a medical decision maker may have to make for you in the future?
	Flexibility	- what it means to give a medical decision maker flexibility to make future decisions?
		- the different amounts of flexibility a person can give their medical decision maker?
	Ask questions	- the types of questions you can ask your doctor that will help you make a good medical decision?
**Contemplation**		**How much have thought about:**
	DM	- who your medical decision maker should be?
		- asking someone to be your medical decision maker?
		- talking with your doctors about who you want your medical decision maker to be?
	QOL	- whether or not certain health situations would make your life not worth living?
		- talking with your decision maker about whether health situations would make your life not worth living?
		- talking with your doctors about whether health situations would make your life not worth living?
	Flexibility	- the amount of flexibility you would want to give your medical decision maker?
		- talking with your medical decision maker about how much flexibility you want to give them?
	Ask questions	- questions you will ask your doctors to help make good medical decisions?
**Self-efficacy**		**How confident are you that today you could:**
	DM	- ask someone to be your medical decision maker?
		- talk with your doctors about who you want your medical decision maker to be?
	QOL	- talk with your decision maker about whether health situations would make your life not worth living?
		- talk with your doctors about whether health situations would make your life not worth living?
	Flexibility	- talk with your decision maker about how much flexibility you want to give them?
	Ask questions	- ask the right questions of your doctors to help make good medical decisions?
**Readiness**		**How ready are you to:**
	DM	- formally ask someone to be your medical decision maker?
		- talk with your doctor about who you want your medical decision maker to be?
		- sign official papers naming a person or group to make medical decisions for you?
	QOL	- talk to your medical decision maker about whether health situations would make life not worth living?
		- talk to your doctor about whether health situations would make your life not worth living?
		- sign official papers about the kind of medical care you would want if you were seriously ill or dying?
	Flexibility	- talk to your medical decision maker about how much flexibility you want to give them?
		- talk to your doctor about how much flexibility you want to give your decision maker?
		- sign official papers about how much flexibility to give your decision maker?
	Ask questions	- ask your doctor questions to help you make a good medical decision?

* Decision Maker (DM), Quality of life and what is most important in life (QOL).

† The knowledge subscale did not ask about QOL as this is a personal determination, not a factual one.

Action Measures assess concrete actions (rather than processes) pertaining to each individual advance care planning behavior, with yes/no response options, such as whether the participant had actually asked someone to be a medical decision maker (DM), whether they had discussed their quality of life with their physician (QOL), whether they wanted to give their surrogate flexibility in decision making (Flexibility), and whether they had asked doctors questions to make a good medical decision (Ask Questions) (Table 2). Therefore, the Action Measures questions were grouped into subscales for each advance care planning behavior domain (DM, QOL, Flexibility, Ask Questions).

**Table 2:** Advance Care Planning Engagement Survey – Action Measures: Advance Care Planning Domains and Questions.

Domain*	Questions	
DM	- Have you already decided who you want your medical decision maker to be?	
	- Have you already formally asked someone to be your medical decision maker?	
	- Have you talked with your doctor about who you want your medical decision maker to be?	
	- Have you signed official papers naming a person or group to make medical decisions for you?	
QOL	- Have you already decided whether or not certain health situations would make your life not worth living?	
	- Have you talked with your decision maker about whether certain health situations would make your life not worth living?	
	- Have you talked with your doctor about whether certain health situations would make your life not worth living?	
	- Have you signed official papers about your wishes for medical care if you were seriously ill or dying in writing?	
Flexibility	- Have you decided how much flexibility to give a medical decision maker if they have to make decisions on your behalf?	
	- Have you talked with your medical decision maker about how much flexibility you want to give her/him?	
	- Have you talked with your doctor about how much flexibility you want to give your medical decision maker?	
	- Have you signed official papers to put your wishes about how much flexibility to give your decision maker in writing?	
Ask Questions		- Have you decided on questions you will ask your doctors to make good medical decisions?
		- Have you ever ask your doctor about the risks of treatment
		- Have you ever ask your doctor about the benefits of treatment
		- Have you ever ask your doctor about your other options to the treatments the doctors were suggesting?
		- Have you ever ask your doctor about what your quality of life would be like after starting a treatment?
		- Have you ever ask your doctor about to repeat information if you did not understand it the first time?

* Decision Maker (DM), Quality of life and what is most important in life (QOL).

When designing the survey, we adhered to health literacy and clear health communication principles for creating written materials for older adults [9]. Not only was plain language used throughout the survey, but the survey was also read to participants verbatim, and participants were given their own copy to follow along that included large text (i.e., 14 point font). We also modified the survey prototype after several cognitive interviews with adults ≥55 years of age or older from the San Francisco VA Medical Center (SFVA) and San Francisco General Hospital (SFGH). We also obtained further input and reviews from the aforementioned content experts. In addition, a preliminary version of the survey was piloted with two separate groups of diverse patients both consisting of a mixture of older adults from the SFVA and SFGH (n = 23, group #1 and n = 62, group #2) to identify questions that needed to be reworded or deleted (data for these pre-tests are not shown).

The Advance Care Planning Engagement Survey used in this study has 31 Process Measure questions. For analysis, we created an overall average Likert score of 1–5 for all Process Measure questions, and average Likert scores of 1–5 for each Process Measure sub-scale (knowledge, contemplation, self-efficacy, and readiness). The survey also includes 18 dichotomous (yes/no) Action Measure questions. For analysis, we created an overall “action scale” ranging from 0–18 (count of yes responses) and subscales for action domains (DM, QOL, Flexibility, Ask Questions).

### Assessment of Reliability and Validity

This field test was performed to examine the reliability of the questions in the Advance Care Planning Engagement Survey by administering it at baseline and one week later to a sample of older individuals residing in the community and nursing homes. One week was chosen to minimize clinical, physical, or psychological life changes that may take place over longer time periods and may otherwise have biased our findings. To evaluate discriminant validity we compared the results of the Advance Care Planning Engagement Survey from the older cohort to a comparison sample of younger healthy individuals. We hypothesized that the younger cohort would have lower scores because they were likely to not have experienced serious illness and medical decision making.

### Participants

We recruited diverse, English-speaking adults aged 55 or older from San Francisco General Hospital, the San Francisco VA Medical Center outpatient clinics and nursing home, and from community health clinics through posted study fliers. This target population was chosen because studies show that most adults become ready to engage in advance care planning in middle to late age or when they are diagnosed with chronic illness [10]. In response to the fliers, potential participants could call study staff or approach staff directly in clinics and senior centers. Participants were eligible if they were not significantly cognitively impaired as assessed by the Short Portable Mental Status Questionnaire (SPMSQ) (score ≥8 out of 10), [11] and did not self-report as being blind (could see well enough to read newspaper print) or deaf (could hear well enough to talk on the phone). For discriminant validity analysis only, and through posted study fliers at local college campuses, we also recruited a comparison group of adults aged 18 to 30 years of age who reported they were healthy, did not have any medical problems, and did not require the care of a regular physician.

To describe participants, we obtained basic demographic information including age; self-reported race/ethnicity; birthplace; religiosity; and social, financial, and health status through self-report. The Control Preferences Scale was used to assess how patients preferred to make their medical decisions with their doctors (i.e., making all decisions on their own, sharing decision making, or to have doctor make decisions) [12]. Previous experience with advance care planning was assessed through self-report yes/no questions such as: “Have you ever filled out an advance directive? Made life threatening medical decisions for yourself? For someone else?” [13] We also included one self-reported, validated question concerning health literacy (limited versus adequate) [14]. We also calculated the mean ± standard deviation (SD) of how long it took for participants to complete the Advance Care Planning Engagement Survey.

### Data analysis

Demographic and other descriptive statistics of both the older cohort and the younger cohort were examined with percentages and means ± standard deviation (SD), including the mean time (± SD, in minutes, that it took participants to complete the Advance Care Planning Engagement Survey.

In the older cohort only, for the Process Measures of behavior change, we assessed internal consistency reliability using Cronbach's alpha at baseline for the overall scale and for each sub-scale of behavior change factors (knowledge, contemplation, self-efficacy, readiness) and calculated 95% confidence intervals (95% CI) [15]. To determine the performance of each question, we also present the observed range of item-scale correlations between individual items and the total overall scale and total subscales, respectively [16]. A Cronbach's alpha of ≥0.70 is generally considered an acceptable level for internal consistency [17]. Cronbach's alpha was not used to assess the dichotomous Action Measures, as each yes/no question was designed to measure discrete advance care planning constructs. In the older cohort only, we also assessed 1 week test-retest reliability with intraclass correlations using Shrout-Fleiss reliability assessments for a fixed set, [18] and calculated 95% CIs [19]. The same rater asked the same questions in the same physical location to the same participants one week later. An intraclass correlation of 0–0.2 indicates poor agreement, 0.3–0.4 indicates fair agreement, 0.5–0.6 indicates moderate agreement, 0.7–0.8 indicates strong agreement; and >0.8 indicates almost perfect agreement [20]. To determine discriminant validity for both the Process and Action Measures, we compared the overall Process Measure and Action Measure scores at baseline between the older cohort and younger cohort using t-tests. We did not perform factor analysis because individual questions and subscales were meant to assess discrete aspects of advance care planning and the analysis would be underpowered based on the current sample size.

We determined a priori that a sample size of 50 would give us ≥ 80% power (two-tailed test at level 0.05) to rule out a Cronbach's alpha less than 0.7 for the overall score (31 items) and each of the subscales (6, 9, 9, and 10 items, respectively) assuming that the true Cronbach's alpha would be at least 0.83 (overall scale) or 0.835 (subscales). Our preliminary work suggested that Cronbach's alpha values were likely to be at least this large. In addition, given an expected interclass correlation coefficient of 0.80, based on preliminary work, and using a two-tailed alpha of 0.05, we determined that a sample size of 50 would give us ≥80% power to rule out a correlation of less than 0.7 for the overall score (18 items) and each of the subscales (4, 4, 4, and 6 items, respectively). For the discriminant validity comparison group of young, healthy individuals, a sample size of 20 would provide greater than 90% power to observe differences of one standard deviation (SD) or more between the younger and older groups. Power was calculated using PASS 2008 (NCSS Software, Kaysville, UT).

## Results

Fifty older adults enrolled (17 from SFGH, 8 from SFVA outpatient clinics, 8 from the SFVA nursing home, and 17 from community clinics in San Francisco) and all subjects completed the baseline and one week assessment. Sample characteristics are shown in Table 3. The group of older participants had a mean age of 69.3 years (SD ±10.5), 38% were women, 42% were non-White, and it took participants a mean of 21.4 minutes (6.2) to complete the survey in person at baseline and 17.9 minutes (±4.9) to complete the survey by phone at 1 week later. Twenty young, healthy individuals from the community were enrolled for the discriminant validity analysis. The group of young participants had a mean age of 23.2 years (±2.7), 50% were women, and 75% were non-White (Table 3).

**Table 3:** Participant Characteristics.

Characteristics	Category	Older Adults	Younger Adults*
		n = 50	n = 20
		No. (%) or	No. (%) or
		mean (SD)	mean (SD)
**Age**	Mean, years (SD)	69.3 (10.5)	23.2 (2.7)
	Range, years	55–92	20–30
**Gender**	Female	19 (38.0)	10 (50.0)
**Race/Ethnicity**	White, Non-Hispanic	29 (58.0)	5 (25.0)
	African American	7 (14.0)	0 (0.0)
	Latino or Hispanic	3 (6.0)	7 (35.0)
	Asian or Pacific Islander	6 (12.0)	7 (35.0)
	Other	5 (10.0)	1 (5.0)
**Education**	≤ high school	8 (16)	0 (0%)
**Religiosity**	Very-to-Extremely	15 (30.6)	0 (0.0)
**Acculturation**	Born out of U.S.	5 (10.4)	3 (15.0)
**Social Support**	Married/long-term relationship	22 (44.9)	0 (0.0)
**Health Literacy**	Limited literacy	14 (28.0)	6 (30.0)
**Health status**	Fair-to-poor health	24 (48.0)	2 (10.0)
**Decision control preferences**	Patient makes decisions	20 (40.0)	9 (45.0)
	Patient-Doctor share decisions	19 (38.0)	9 (45.0)
	Doctor makes decisions	11 (22.0)	2 (10.0)
**Prior care planning**	Complete advance directive	24 (48.0)	1 (5.0)
	Life/death decisions for self	22 (44.0)	0 (0.0)
	Life/death decisions for others	20 (40.0)	3 (15.0)
**Time to complete survey**	Baseline: Mean minutes (SD)	21.4 (6.2)	12.8 (2.1)
	One week: Mean minutes (SD)	17.9 (4.9)	12.0 (1.3)

* Used as a comparison group for discriminant validity only.

For the older adults only, the overall mean Likert for the Process Measures was 3.7 (±0.7) on a 5-point scale. The overall Cronbach's alpha was 0.94 and was 0.84 for the subscale of knowledge, 0.86 for contemplation, 0.83 for self-efficacy, and 0.92 for readiness (Table 4). The range of item-scale correlations for the total Process Measures score was 0.24 to 0.77. The one question driving the low item-scale correlation was “How well informed are you about who can be a medical decision maker?” in the knowledge subscale. However, deleting this question did not appreciably change the Cronbach's alpha for the overall Process Measure score or the individual knowledge score, so we retained this question because it is clinically important. The 1-week test-retest intraclass correlation for the overall Process Measures was 0.70, and was 0.70 for the behavior change factor subscale of knowledge, 0.56 for contemplation, 0.60 for self-efficacy, and 0.69 for readiness.

**Table 4:** Advance Care Planning Engagement Survey – Process Measures: Reliability, Item-Scale Correlations, & Descriptive Statistics.

	# of items	Mean* +/−SD	Cronbach's Alpha (95% CI)†	Range item-scale correlations	Intra-class correlations‡
**Total Process Measure Score**	31	3.7 (0.7)	0.94 (0.91–0.96)	0.24–0.77	0.70 (0.54–0.82)
**Behavior Change Subscales**					
Knowledge‡	6	3.5 (0.7)	0.84 (0.76–0.90)	0.39–0.69	0.70 (0.54–0.82)
Contemplation	9	3.4 (0.9)	0.86 (0.79–0.91)	0.43–0.75	0.56 (0.37–0.73)
Self-efficacy	6	3.9 (0.7)	0.83 (0.75–0.89)	0.41–0.74	0.60 (0.41–0.76)
Readiness	10	4.0 (0.9)	0.92 (0.88–0.95)	0.60–0.76	0.69 (0.53–0.81)

* Mean Scale Score based on average of items in each scale scored on 1–5Likert scale.

† Raw Cronbach's Coefficient Alpha calculated. Mean Scale Score based on average Likert score.

‡ Interaclass correlations were calculated using Shrout-Fleiss reliability assessments for a fixed set.

The overall mean score for the Action Measures was 10.1 (±3.6) on an 18-point scale. The overall intraclass correlation was 0.87 and was 0.81 for the action items related to the advance care planning domains of decision makers, 0.87 for quality of life, 0.83 for flexibility, and 0.57 for asking doctors questions (Table 5).

**Table 5:** Advance Care Planning Engagement Survey – Action Measures: Reliability, & Descriptive Statistics.

	# of items	Mean Score† (+/−SD)	Intra-class correlations (95% CI)‡
**Total Action Measure Score**	18	10.1 (3.6)	0.87 (0.79–0.92)
**Action Subscales**			
DM*	4	2.2 (1.1)	0.81 (0.70–0.89)
QOL	4	1.8 (1.4)	0.87 (0.79–0.92)
Flexibility	4	1.7 (1.3)	0.83 (0.72–0.90)
Ask Questions	6	4.4 (1.7)	0.57 (0.38–0.74)

* Decision Maker (DM) and Quality of life and what is most important in life (QOL).

† Means scores range from 0–18 for the Total Action Score, to 0–4 for Decision Maker, QOL, and Flexibility, and 0–6 For Ask Questions.

‡ Calculated using Shrout-Fleiss reliability assessments for a fixed set.

For discriminant validity of the Process Measures, younger healthy adults had an average overall Likert score of 2.7 (±0.8) versus 3.7 (±0.7) in the older cohort (37% higher), p<0.001. For discriminant validity of the Action Measures, the younger healthy adults had an average total mean Action Measure score of 5.3 (±2.5) versus 10.1 (±3.6) in the older cohort (91% higher), p<0.001.

## Discussion

We developed and examined the reliability and discriminant validity of a new Advance Care Planning Engagement Survey which measures both behavior change Process Measures (quantifies knowledge, contemplation, self-efficacy, and readiness) and Action Measures concerning engagement in multiple advance care planning actions defined by prior research and our conceptual model to include the domains of surrogate decision makers (DM), values and quality of life (QOL), flexibility for the surrogate (Flexibility), and asking doctors questions (Ask Questions). The individual Process Measure subscales of knowledge, contemplation, self-efficacy, and readiness, and the individual Action Measure subscales of DM, QOL, Flexibility, and Asking Questions also performed well, and may be able to be used on their own.

This study also confirms findings from other research that participants are engaging not just in advance directive completion, but also in a wide range of advance care planning behaviors, such as discussions with surrogates and clinicians, which may be equally helpful for end-of-life decision making [4], [8]. Additionally, people are in several different stages of behavioral change for each of these behaviors, as has been found in other studies [3]. Based on our findings, prior advance care planning interventions studies may have grossly underestimated their impact by solely focusing on advance directive completion and not on the processes of behavior change and multiple advance care planning behaviors. For example, a study of the impact of the Terri Schiavo media coverage found that her story motivated 61% of English and Spanish-speaking older adults to define their own goals for medical care and 66% to talk to their family and friends about advance care planning, yet only 3% completed an advance directive [21]. Terri Schiavo's story clearly had a strong impact on this patient population. However, if only advance directive completion was assessed, this would have been considered a negative study.

To our knowledge, Fried and colleagues have been the only other researchers to validate surveys focused on behavior change in advance care planning. One survey includes global questions concerning decisional balance, medical and religious beliefs, and processes of change for advance care planning in general, and another assesses stage of behavior change for several advance care planning behaviors including completing advance directive forms and talking with surrogates and physicians [3], [22]. The current Advance Care Planning Engagement Survey expands this range of advance care planning behaviors to include other behaviors identified in prior research and in our conceptual model such as flexibility in surrogate decision making and learning how to ask doctors questions to make informed medical decisions. The Advance Care Planning Engagement Survey also systematically quantifies several behavior change factors (knowledge, contemplation, self-efficacy, and readiness) for each individual advance care planning behavior using 5-point Likert response options. This approach may provide greater power to observe small, yet clinically meaningful, differences in behavior change in response to an advance care planning intervention. One great benefit of Fried's survey is the ability to classify individuals into specific behavior change stages, such as pre-contemplation, contemplation, preparation, action, and maintenance. To capture the full process of advance care planning, investigators may consider using a combination of all 3 surveys.

### Limitations

It is important to note that this study was conducted in one region of the country and participants were recruited through convenience sampling, which may compromise generalizability. We attempted to minimize this concern by recruiting a diverse sample of elders (42% Hispanic or non-White, 38% female, 28% with limited health literacy). In addition, the study also has a small sample size. However, the robust results suggest that power did not play a large factor in determining validation of the survey. The younger, healthy comparative group used to determine discriminant validity was not matched on gender or race with the older cohort, which may have affected our results. It is also important to note that the Action Measure subscale of Asking Doctors Questions had moderate ICC agreement that was slightly lower than the other subscales. Because this question asked about the risks, benefits, and other options for treatment, it is possible that individuals learned about the questions from time 1 to time 2. It is also possible that this subscale may not be as robust for use on its own. We also acknowledge that the survey took an average of 20 minutes to complete, which may limit its usefulness in some studies. However, shorter surveys may not be able to fully assess the complex construct of behavior change and advance care planning. Further studies will be needed to determine whether the survey can detect clinically important changes over time.

### Conclusion

In conclusion, a new Advance Care Planning Engagement Survey that measures advance care planning behavior change demonstrates good reliability and discriminant validity in this field test. Because the individual Process Measures (knowledge, contemplation, self-efficacy, and readiness) as well as Action Measures of a broad complement of advance care planning actions performed well, individual components of the survey may be able to be used on their own in other studies. Further research is needed to assess whether the Advance Care Planning Engagement Survey scores improve in response to an advance care planning intervention and whether survey scores are associated with satisfaction in care, and receipt of medical care that is consistent with one's values.
